# Development and validation of a deep learning–based assessment tool for teacher leadership: A case study from Xinjiang, China

**DOI:** 10.1371/journal.pone.0331560

**Published:** 2025-09-02

**Authors:** Jianwei Dong, Xinya Chen, Chen Chen, Cheng Chen

**Affiliations:** 1 College of Education Science, Xinjiang Normal University, Urumqi, China; 2 College of Education Science, Xinjiang Teacher’s College, Urumqi, China; 3 College of Information Science and Engineering, Xinjiang University, Urumqi, China; 4 College of Software, Xinjiang University, Urumqi, China; 5 Department of Cardiology, People’s Hospital of Xinjiang Uyghur Autonomous Region, Urumqi, China; 6 Xinjiang Key Laboratory of Cardiovascular Homeostasis and Regeneration Research, Urumqi, China; Prince Sattam bin Abdulaziz University, SAUDI ARABIA

## Abstract

Teacher leadership is widely regarded as a critical driver of school reform and educational quality improvement. Although the field has been extensively studied, empirical research remains limited in Xinjiang, China—a region characterized by its multiethnic and multilingual context. To address this gap, the present study developed and validated a culturally sensitive assessment tool based on a sample of 371 primary and secondary school teachers from Xinjiang. A structured questionnaire was designed encompassing four dimensions: professional guidance, educational collaboration, cross-cultural ICT-based teaching competence, and leadership cognition. In addition, we introduced an interpretable deep learning model—ITL-LSTM (Interpretable Teacher Leadership LSTM)—which employs a Diagonal BiLSTM structure for dynamic classification of teacher leadership profiles, achieving a prediction accuracy of 90.10%. The findings indicate that the proposed tool demonstrates strong applicability and scalability within the Xinjiang context, providing effective support for dynamic evaluation, personalized development, and evidence-based decision-making in multicultural educational settings.

## 1. Introduction

Among the numerous key elements in the field of education, teacher leadership has a significant and non – negligible impact on the development process of schools and the growth trajectory of students [1–4]. In recent years, with the continuous deepening of educational research, teacher leadership has become a key issue of common concern in both academic and educational practice fields [5,6]. A large number of studies have been carried out around its promoting effects on teaching quality improvement, teacher professional growth, and students’ academic achievements, providing rich theoretical and practical experience for educational development [7–9].

However, compared with the developed eastern regions, research on teacher leadership in border areas, especially in Xinjiang, is still weak [10]. In Xinjiang, China, due to its unique social background of multi – ethnic settlement and multi – cultural integration, research on teacher leadership is relatively scarce. The educational environment in Xinjiang has distinct particularities. The cultures of multiple ethnic groups collide and integrate on campus, which places higher demands on teachers’ leadership abilities [11–13]. But existing studies have failed to comprehensively and deeply reveal the actual situation of teacher leadership in primary and secondary schools in Xinjiang. There is a lack of systematic analysis of various factors that affect the exertion of teacher leadership, such as ethnic and cultural differences, regional education, and unbalanced educational resources. Moreover, the development path of teacher leadership that conforms to local characteristics has not been explored yet. There is a lack of in – depth discussion on how to further enhance teacher leadership by combining the rich cultural characteristics of Xinjiang to improve the quality of education and teaching, which has a significant gap with the urgent need for high – quality education in Xinjiang.

At the same time, with the development of artificial intelligence technology, the application of deep learning in educational assessment and decision – making support is becoming more and more extensive. Introducing deep – learning models into teacher leadership research not only helps to improve the accuracy and scientific nature of analysis but also provides a new path for the intelligent assessment of teacher capabilities [14–16].

Although various influential definitions, models, and assessment frameworks for teacher leadership have been proposed, most existing studies are concentrated in economically developed regions and lack empirical applicability to areas with distinct social structures and educational ecologies such as Xinjiang. This study aims to address the contextual complexity and cultural diversity of the education system in Xinjiang, China, by developing an adaptive and interpretable assessment tool for teacher leadership. By constructing a four-dimensional scale—comprising professional guidance, educational collaboration, cross-cultural ICT-based teaching competence, and leadership cognition—and integrating it with a Diagonal BiLSTM deep learning model, this study enables dynamic identification and predictive analysis of teacher leadership. The goal is to establish a scalable assessment framework applicable to multiethnic, resource-constrained educational settings, thereby supporting teacher development and informing education policy and practice.

## 2. Review

The theory of teacher leadership originated from the education reform in the United States in the 1980s, emphasizing the crucial role of teachers in teaching improvement and school development. With the in – depth study, this theory has gradually shifted from the understanding of “position power” to that of “professional influence” and “collective participation” [3,17]. Currently, teacher leadership is widely defined as the comprehensive ability of teachers to have a positive impact on the educational environment through knowledge, skills, emotions, and collaborative behaviors [18,19]. It emphasizes the leadership role of teachers in promoting teaching quality, driving school change, and constructing a professional culture, especially in cross – hierarchical interactions in formal and informal fields [20].

In recent years, international research on teacher leadership models has become more systematic. The “Teacher Leader Model Standards” and the “Teacher Leadership Framework” released by the Teacher Leadership Exploratory Consortium in the United States have further clarified its core dimensions, including self – development, innovation leadership, coaching ability, and teamwork [2]. These studies have promoted the transformation of teacher leadership from theory to practice and provided a clear path for education reform.

In China, especially in multi – ethnic regions such as Xinjiang, research on teacher leadership has gradually attracted attention. Existing studies have shown that teacher leadership styles directly affect students’ academic achievements and learning environments, and play an important role in constructing educational equity, cultural integration, and teacher professional growth [21,22]. For example, research on Shanghai’s PISA scores has emphasized the role of teacher leadership in curriculum implementation and teacher team construction, and empirical analysis in Beijing also supports its positive effects on academic performance and campus culture construction.

Overall, the core attributes of teacher leadership include ethicality, professionalism, and collectivism [23]. Its essence is an internally – driven force for educational change. Especially in regions with diverse social cultures and complex educational challenges, it has greater research and practical value. Based on this, this article aims to explore the performance characteristics and development mechanisms of teacher leadership in Xinjiang, providing theoretical support and practical paths for the high – quality development of education in border areas.

### 2.1. Existing teacher leadership assessment tools and their limitations

**Table:** 

Author, Year	Instrument Name	Dimensions Measured	Sample/Region	Methodology	Limitations
**Pazur, 2022** [24]	**Democratic School Leadership Characteristics Scale**	**Teacher participation, collaborative climate, and other democratic leadership traits**	**651 teachers in Croatia**	**Expert review, principal component analysis**	**Single-dimensional; limited regional generalizability**
**Chen, 2022** [25]	**Teacher Leadership Inventory (TLI)**	**Instructional leadership, professional development, collaboration, school improvement, diverse influence**	**477 secondary school teachers in Mainland China**	**Questionnaire, structural equation modeling**	**Static measurement; lacks dynamic behavioral analysis**
**Eckert et al., 2022** [26]	**Collective Leadership Measurement Tool**	**Readiness, efficacy, and team effectiveness in collective leadership**	**Teacher groups from various U.S. schools**	**Multi-group confirmatory factor analysis (MGCFA)**	**Static instrument; does not account for dynamic characteristics**
**Darwis et al., 2025** [27]	**Potential Leadership Assessment Tool for Principals**	**Learning vision, culture, environment commitment, advocacy, strategic vision, resource management, collaboration, instructional leadership, partnerships**	**117,174 primary school principals and teachers in Indonesia**	**Hinkin-based instrument development model, expert review, interviews, questionnaire**	**Region-specific; lacks capacity to capture dynamic leadership behaviors in diverse environments**
**Zhang et al., 2025** [28]	**Sustainable Leadership Scale for University Teachers**	**Purpose and values, teaching strategies, research capacity, collaborative dialogue, operational capacity**	**1,108 faculty members from 10 Chinese universities**	**EFA + CFA + reliability & validity tests; AMDSL-HEI framework for higher ed**	**Focused on higher education; lacks applicability to basic education and cross-cultural contexts**
**This Study (2025)**	**ITL-LSTM + Xinjiang Teacher Leadership Scale**	**Professional guidance, educational collaboration, cross-cultural digital competence, leadership cognition**	**371 multi-ethnic primary and secondary school teachers in Xinjiang**	**Four-dimensional scale (Cronbach α = 0.899) + Diagonal BiLSTM deep learning (accuracy = 90.10%)**	**—**

A comparative analysis of the five aforementioned studies reveals several common limitations among existing teacher leadership assessment tools:

Static measurement models lacking dynamic behavior capture: Instruments such as Chen’s (2022) Teacher Leadership Inventory (TLI) and Eckert’s (2022) Collective Leadership Tool primarily rely on structural equation modeling and multi-group confirmatory factor analysis (MGCFA). These approaches are inherently static and fail to reflect the evolving nature of teacher leadership in real-world educational settings.

Limited applicability across regions and populations: Pazur’s (2022) Democratic Leadership Scale is confined to the Croatian context, while Darwis et al.’s (2025) tool for Indonesian principals is similarly restricted to a single national setting. Both fail to account for the dynamic adaptability required in multicultural and diverse educational environments. Although Zhang et al. (2025) employed a large university sample in China, their focus on higher education excludes applicability to K–12 settings and cross-cultural factors.

Lack of integration with advanced technologies: Most existing tools remain grounded in traditional quantitative analysis, with minimal incorporation of artificial intelligence or deep learning methods. As such, they fall short in accurately predicting or explaining the dynamic trajectories of teacher leadership development.

## 3 Materials and methods

### 3.1 Research method

Therefore, this study aims to develop and validate a teacher leadership assessment tool tailored to the educational complexities of China’s Xinjiang region, characterized by its multi-ethnic and multilingual context. Based on construct validity principles, we designed the “Teacher Leadership Survey for Primary and Secondary Schools,” incorporating four key dimensions: Professional Guidance, Educational Collaboration, Cross-cultural Digital Instructional Competence, and Leadership Cognition. A reliability and validity analysis was conducted on a sample of 371 responses to ensure the scientific soundness and contextual applicability of the scale structure. Building upon this, we further introduced an interpretable deep learning model—ITL-LSTM—based on a Diagonal BiLSTM architecture, enabling dynamic classification and prediction of teacher leadership levels. The model provides an intelligent, interpretable, and generalizable evaluation tool for educational systems operating under complex cultural conditions [29].

### 3.2 Data collection

The research tool used in this study is the “Questionnaire on the Current Situation of Leadership among Primary and Secondary School Teachers in Xinjiang Uygur Autonomous Region”, which was compiled by experts in the field of education and has high content validity. This questionnaire consists of two parts. The first part is a survey of basic information about teachers, including variables such as gender, age, ethnicity, years of teaching experience, subjects taught, educational levels, professional titles, and school regions.

The second part is the Scale of Leadership for Primary and Secondary School Teachers, which comprises four dimensions: professional guidance, educational collaboration, leadership cognition, and cross-cultural information technology teaching ability. To avoid potential expectancy biases of the respondents regarding the four dimensions, the dimension divisions were not explicitly stated during the formal survey process. After the questionnaires were collected, the scores of the four dimensions were calculated by categorizing the items [30]. The scale uses the Likert five-point scale method for measurement, with 5 representing complete compliance, 4 representing relatively compliance, 3 representing basic compliance, 2 representing basic non-compliance, and 1 representing complete non-compliance.

### 3.3 Implementation of the survey

This study distributed the “Survey Questionnaire on the Current Situation of Leadership among Primary and Secondary School Teachers in Xinjiang Uygur Autonomous Region” to primary and secondary schools in Xinjiang. A total of 400 questionnaires were collected(The dataset is shown in S1 Data). The survey data were cleaned and analyzed using SPSS 26.0 statistical analysis software. Among them, there were 29 cases where respondents consistently chose the same answer or continuously provided agreement responses. These cases were cleaned, resulting in a final sample of 371 valid questionnaires. The specific statistical details are presented in Table 1.

**Table 1:** Basic Information of Survey Participants.

Basic Information	Category	Quantity	Percentage
Gender	Female	280	75.7%
	Male	91	24.6%
Age	20-30 years	77	20.8%
	31-40 years	136	36.8%
	41-50 years	123	33.2%
	51 years and above	35	9.5%
Ethnicity	The Han nationality	277	74.9%
	The Uyghur	42	11.4%
	The Kazakh	29	7.8%
	Other ethnicities	23	6.2%
Teaching Experience	5 years and below	74	20.0%
	6-10 years	70	18.9%
	11-20 years	121	32.7%
	20 years and above	106	28.6%
Education	College	42	11.4%
	Bachelor’s degree	297	80.3%
	Master’s degree	31	8.4%
	Doctoral degree	1	0.3%
Region	Urumqi City	115	31.1%
	Xinjiang Production and Construction Corps	4	1.1%
	Northern Xinjiang	176	47.6%
	Southern Xinjiang	71	19.2%
	Eastern Xinjiang	5	1.4%
Graduated from Normal University	yes	307	83.0%
	no	64	17.3%
Educational Stage	Primary School	146	39.3%
	Junior High School	207	55.8%
	High School	41	11.1%
	Vocational School	6	1.6%
Professional Title	None	33	8.9%
	Junior	103	27.8%
	Intermediate	142	38.4%
	Senior	91	24.6%
	Chief	2	0.5%

From the perspective of questionnaire completion, our survey covers a wide range of population and regional distribution, exhibiting certain characteristics specific to Xinjiang. The Han ethnic group accounts for 74.9% of the total population, while Uyghurs account for 11.4%, Kazakhs and other ethnic minorities account for 7.8% and 6.2%, respectively, in line with the distribution proportion of ethnic minorities in Xinjiang. Northern Xinjiang accounts for 47.6% of the total population, while Urumqi and Southern Xinjiang account for 31.1% and 19.2%, respectively, occupying a relatively large proportion in the overall distribution. Eastern Xinjiang and the Xinjiang Production and Construction Corps have relatively fewer numbers, accounting for 1.4% and 1.1%, respectively. In terms of educational stages, our research mainly focuses on primary and secondary schools, with junior high schools accounting for the largest proportion at 55.8%, and primary schools accounting for 39.3%.

### 3.4. Project analysis

The item analysis method is widely used in assessing the discriminative power of scale items. In this study, using SPSS 26.0 software, we calculated the total scores of the respondents on the teacher leadership scale to measure their leadership capabilities. Based on this analysis, we categorized the participants into two groups: a high-scoring group and a low-scoring group. Specifically, participants scoring in the top 27% were classified as the high-scoring group, while those scoring in the bottom 27% were classified as the low-scoring group.

To further explore the discriminative power of each item between the two groups, independent samples t-tests were conducted. Table 2 showed that all sixteen items exhibited significant discriminative power between the high-scoring and low-scoring groups (P < 0.05). Specifically, the scores of the high-scoring group were significantly higher than those of the low-scoring group, indicating that these items possess good discriminative power in assessing the leadership level of the surveyed teachers and successfully passed the item analysis test.

**Table 2:** Item Analysis for Each Question.

Title	Group	Number of Cases	Mean	t-value	Sig.
1. I have a clear plan for my future professional development.	High	100	4.41	11.066	0.000
	Low	100	3.33		
2. In my own professional development, I need to enhance myself through training, peer communication, and other means.	High	100	4.75	9.610	0.000
	Low	100	3.80		
3. I believe that research topics are important and can change the way education and teaching are conducted.	High	100	4.58	8.492	0.000
	Low	100	3.59		
4. I prioritize leading the way in information technology and encourage students to appropriately obtain practical resources online.	High	100	4.70	11.643	0.000
	Low	100	3.73		
5. I try to lead and effectively interact with individuals from different backgrounds, ethnicities, and cultures simultaneously.	High	100	4.77	13.201	0.000
	Low	100	3.76		
6. I value the cultivation of Chinese traditional culture in students and emphasize the development of their multicultural abilities.	High	100	4.89	14.802	0.000
	Low	100	4.00		
7. I foster a culture of trust, inclusiveness, and reflection, encouraging different viewpoints to clash with each other.	High	100	4.89	15.053	0.000
	Low	100	3.94		
8. I have clear goals and strategies for home-school cooperation, achieving effective interaction between home and school.	High	100	4.84	16.800	0.000
	Low	100	3.68		
9. I understand the backgrounds of different students and strive to ensure fair development for all students.	High	100	4.87	14.812	0.000
	Low	100	3.81		
10. I often discuss various educational issues with colleagues and communicate with parents.	High	100	4.89	20.021	0.000
	Low	100	3.84		
11. I frequently communicate with parents to jointly solve various issues related to student development.	High	100	4.74	14.085	0.000
	Low	100	3.77		
12. I strive to garner more assistance and resources from parents and communities to achieve the school’s development goals.	High	100	4.76	14.280	0.000
	Low	100	3.57		
13. My words and actions can influence changes in parents.	High	100	4.69	14.113	0.000
	Low	100	3.50		
14. I have a very good understanding of teacher leadership.	High	100	4.40	11.400	0.000
	Low	100	3.25		
15. I possess teacher leadership.	High	100	4.35	10.864	0.000
	Low	100	3.25		
16. Teacher leadership behaviors are present in my school.	High	100	4.27	7.650	0.000
	Low	100	3.37		

### 3.5. Reliability analysis

Reliability refers to whether the questionnaires in the research sample are truly reliable and whether the statistical data align with the actual situations in natural contexts. In this study, reliability was examined by calculating the values of Cronbach’s alpha coefficient and split-half reliability coefficient to systematically assess the internal consistency of the Teacher Leadership Scale and verify whether the questionnaire has good reliability.

#### 3.5.1. Cronbach’s alpha coefficient analysis.

Cronbach’s alpha coefficient is the most widely used tool for evaluating reliability in current research According to the views of the American scholar Kline, when the alpha coefficient is below 0.5, the reliability analysis is unacceptable, while when it is above 0.5, the reliability analysis is acceptable. Additionally, alpha coefficients greater than 0.5 are further categorized into several degrees: moderate reliability when α > 0.7, good reliability when α > 0.8, and optimal reliability when α > 0.9. In other words, the higher the alpha coefficient, the better the reliability, indicating a more stable internal structure of the scale.

In this study, Cronbach’s alpha coefficient was calculated for the Teacher Leadership Scale developed for primary and secondary school teachers. As shown in Table 3, the alpha coefficient for the overall Teacher Leadership Scale is 0.899, indicating good reliability of the overall structure of the scale and the teacher leadership structure having good internal consistency.

**Table 3:** Cronbach’s Alpha Values for the Scale.

Scale	Number of Items	Cronbach’s Alpha Coefficient
Teacher Leadership Scale for Primary and Secondary Schools	16	0.899

#### 3.5.2. Split-half reliability coefficient analysis.

The split-half reliability coefficient, also known as the Spearman-Brown coefficient, is another important tool for assessing reliability. In this study, the Spearman-Brown split-half coefficient for the Teacher Leadership Scale was calculated. The specific results are shown in Table 4, indicating a split-half coefficient of 0.834, which is greater than 0.8. This suggests that the structure of the scale has good reliability.

**Table 4:** Spearman-Brown Split-Half Reliability Coefficient Values.

Scale	Number of Items	Spearman-Brown Split-Half Reliability
Teacher Leadership Scale for Primary and Secondary Schools	16	0.834

In summary, through the dual examination of Cronbach’s alpha coefficient and split-half reliability coefficient values, it was found that the Teacher Leadership Scale for primary and secondary school teachers has good reliability. This indicates that the research data obtained are highly credible, and the structure exhibits internal consistency.

### 3.6. Validity analysis

In the academic research process, to verify the effectiveness of measurement dimensions, two statistical tools are commonly relied upon: the Kaiser-Meyer-Olkin (KMO) measure and Bartlett’s test of sphericity. The KMO measure is specifically used to assess the correlations between variables. A value closer to 1 indicates stronger correlations between variables, facilitating subsequent factor analysis. Bartlett’s test of sphericity, on the other hand, is used to determine whether the observed data’s correlation matrix is an identity matrix, thereby assessing whether the data are suitable for factor analysis.

According to commonly accepted research standards domestically and internationally, the KMO measure should exceed 0.7, and Bartlett’s test of sphericity significance coefficient should be less than 0.05 to meet statistical requirements, ensuring that the dimensions being tested have good validity. Only when these two conditions are simultaneously met can we conclude that the effectiveness of the measurement tool has been effectively verified, providing solid support for subsequent research.

By examining the data in Table 5, we can observe that the KMO statistic is 0.914, significantly higher than the threshold of 0.7, indicating strong correlations between variables. Additionally, Bartlett’s test of sphericity yields a chi-square value of 2892.572, with a significance P-value of 0.000, significantly less than the 0.05 significance level. This implies that the correlation matrix of the data is not an identity matrix and is highly suitable for factor analysis. Therefore, we can conclude that the item validity of the scale used in this study is excellent, and the data quality fully meets the requirements for factor analysis.

**Table 5:** KMO and Bartlett’s Test.

Measure of Sampling Adequacy (KMO)		0.914
Bartlett’s Test of Sphericity	Approx. Chi-Square	2892.572
	Degrees of Freedom	120
	Significance	0.000

Given that the Teacher Leadership Scale used in this study was developed jointly by an expert team, exploratory factor analysis was conducted using SPSS software to further ensure the structural validity of the scale. Through principal component analysis, four common factors were successfully extracted, as indicated in Table 6 below. These four common factors contributed to the explanation of variances at rates of 43.313%, 8.520%, 7.938%, and 6.262%, respectively, collectively accounting for 66.033% of the total variance explained, meeting the requirement in academic research for the total variance explained to exceed 60%.

**Table 6:** Total Variance Explained.

Component	Initial Eigenvalues			Extraction Loadings Squared Sum			Rotation Loadings Squared Sum		
	Total	Variance Percentage	Cumulative Percentage	Total	Variance Percentage	Cumulative Percentage	Total	Variance Percentage	Cumulative Percentage
1	6.930	43.313	43.313	6.930	43.313	43.313	4.525	28.280	28.280
2	1.363	8.520	51.834	1.363	8.520	51.834	2.125	13.279	41.559
3	1.270	7.938	59.771	1.270	7.938	59.771	2.008	12.549	54.109
4	1.002	6.262	66.033	1.002	6.262	66.033	1.908	11.925	66.033
5	0.764	4.778	70.811						
6	0.675	4.217	75.028						
7	0.634	3.960	78.988						
8	0.543	3.392	82.380						
9	0.488	3.049	85.429						
10	0.460	2.875	88.304						
11	0.434	2.713	91.018						
12	0.364	2.272	93.290						
13	0.311	1.943	95.232						
14	0.298	1.864	97.096						
15	0.292	1.827	98.923						
16	0.172	1.077	100.000						

Extraction Method: Principal Component Analysis.

After factor rotation using the maximum variance method, the variance explained ratios of the four common factors changed to 28.280%, 13.279%, 12.549%, and 11.925%, respectively. Although the specific contribution rates of each common factor were adjusted, the total variance explained remained stable at 66.033%. This result further confirms the stability and rationality of the scale structure.

The first factor extracted using the maximum variance method includes the following items: “I foster a culture of trust, inclusiveness, and reflection, encouraging the exchange of different viewpoints,” “I have clear goals and strategies for cooperation between home and school, facilitating effective interaction,” “I understand students from diverse backgrounds and strive to ensure their equitable development,” “I frequently discuss various educational issues with colleagues and communicate with parents,” “I make efforts to garner more assistance and resources from the parent community to achieve school development goals,” and “My words and actions can influence changes in parents.” The factor loadings for each item exceed 0.5. Therefore, we label this factor as “Educational Synergy.” Educational Synergy mainly reflects teachers’ leadership in communication between home and school and social influence. It encompasses various efforts such as fostering a culture of trust, setting clear cooperation goals, addressing individual student differences, effective communication with colleagues and parents, and leveraging personal influence to contribute to students’ comprehensive development and the sustainable development of schools.

The second factor extracted after rotation using the maximum variance method comprises three items: “I emphasize leading students in information utilization, encouraging appropriate online resource utilization,” “I attempt to guide students from different backgrounds and cultures to interact effectively,” and “I attach importance to cultivating students’ understanding of Chinese traditional culture and actively foster their multicultural capabilities.”

The factor loadings for these three items all exceed 0.5, indicating their significant contribution to the second factor. Given the characteristics of these items, we name this factor “Cross-cultural Information Teaching Capability.” This factor primarily reflects teachers’ leadership in using informational tools for cross-cultural teaching. It demonstrates that teachers, in the era of information technology, need not only traditional teaching skills but also leadership in fostering students’ cross-cultural awareness and adaptability to address the challenges of globalization in education.

Through factor rotation using the maximum variance method, we successfully extracted a third crucial factor. This factor encompasses three specific items: “I have a very good understanding of teacher leadership,” “I possess teacher leadership,” and “Teacher leadership behaviors are present in my school..” The factor loadings for all three items exceed 0.5. Based on the core attributes of these items, we label this factor as “Leadership Cognition.” This factor not only reveals teachers’ self-perception of their leadership abilities but also reflects their general awareness of teacher leadership behavior within the school environment.

Finally, after factor rotation using the maximum variance method, we identified a fourth significant factor. This factor consists of three specific items: “I have a clear plan for my future professional development,” “I need to enhance myself through training and peer exchange in my professional development,” and “I believe that research projects are essential and can change educational teaching methods through research.” The factor loadings for these three items all exceed 0.5, indicating their substantial contribution to this factor. Given the shared characteristics of these items, we label this factor as “Teacher Guiding Capacity.” The rotated component matrix is presented in Table 7.

**Table 7:** Rotated Component Matrix.

Title	Component			
	1	2	3	4
1. I have a clear plan for my future professional development.				0.563
2. In my own professional development, I need to enhance myself through training, peer communication, and other means.				0.772
3. I believe that research topics are important and can change the way education and teaching are conducted.				0.819
4. I prioritize leading the way in information technology and encourage students to appropriately obtain practical resources online.		0.763		
5. I try to lead and effectively interact with individuals from different backgrounds, ethnicities, and cultures simultaneously.		0.796		
6. I value the cultivation of Chinese traditional culture in students and emphasize the development of their multicultural abilities.		0.616		
7. I foster a culture of trust, inclusiveness, and reflection, encouraging different viewpoints to clash with each other.	0.649			
8. I have clear goals and strategies for home-school cooperation, achieving effective interaction between home and school.	0.763			
9. I understand the backgrounds of different students and strive to ensure fair development for all students.	0.773			
10. I often discuss various educational issues with colleagues and communicate with parents.	0.836			
11. I frequently communicate with parents to jointly solve various issues related to student development.	0.800			
12. I strive to garner more assistance and resources from parents and communities to achieve the school’s development goals.	0.740			
13. My words and actions can influence changes in parents.	0.635			
14. I have a very good understanding of teacher leadership.			0.828	
15. I possess teacher leadership.			0.753	
16. Teacher leadership behaviors are present in my school.			0.613	

Extraction Method: Principal Component Analysis. Rotation Method: Kaiser Normalization with Varimax Rotation. Convergence was achieved after 6 iterations.

### 3.7. Analysis of scores on the five-dimensional scale

As indicated in Table 8, the overall average score for leadership among primary and secondary school teachers in Xinjiang is 4.099 points. Among the five dimensions, the highest average score is for Cross-cultural Information Teaching Capability, at 4.238 points, while the average score for Leadership Cognition is the lowest, at 3.830 points. Overall, both the total leadership score and the average scores for the four dimensions are above 4 points, indicating a favorable overall situation of leadership among primary and secondary school teachers in Xinjiang.

**Table 8:** Detailed Scores of Leadership Levels among Primary and Secondary School Teachers.

Factor	Number of Items	Minimum Value	Maximum Value	Average Score	Standard Deviation
Professional Guidance		1.33	5.00	4.043	0.589
Educational Collaboration		2.29	5.00	4.177	0.490
Leadership Cognition		1.00	5.00	3.830	0.601
Cross-cultural Information Technology Teaching Ability		2.00	5.00	4.238	0.507
Total Leadership	16	2.38	5.00	4.099	0.424

Table 9 provides a detailed display of the scores for each item in the four-dimensional scale, revealing the cognition and emphasis of primary and secondary school teachers on different aspects. Among them, the item “In my own professional development, I need to improve myself through training and peer exchange” in the dimension of “Professional Guidance” scored the highest at 4.23 points. This result indicates that primary and secondary school teachers generally place great importance on opportunities for their professional development and are eager to enhance their professional competence through training and exchange.

**Table 9:** Detailed Scores of Leadership Levels among Primary and Secondary School Teachers.

Dimension	Specific Content	Score	Standard Deviation
Professional Guidance	1. I have a clear plan for my future professional development.	3.88	0.734
	2. In my own professional development, I need to enhance myself through training, peer communication, and other means.	4.23	0.700
	3. I believe that research topics are important and can change the way education and teaching are conducted.	4.02	0.838
Cross-cultural Information Technology Teaching Ability	4. I prioritize leading the way in information technology and encourage students to appropriately obtain practical resources online.	4.17	0.623
	5. I try to lead and effectively interact with individuals from different backgrounds, ethnicities, and cultures simultaneously.	4.19	0.626
	6. I value the cultivation of Chinese traditional culture in students and emphasize the development of their multicultural abilities.	4.36	0.568
Educational Collaboration	7. I foster a culture of trust, inclusiveness, and reflection, encouraging different viewpoints to clash with each other.	4.28	0.566
	8. I have clear goals and strategies for home-school cooperation, achieving effective interaction between home and school.	4.18	0.614
	9. I understand the backgrounds of different students and strive to ensure fair development for all students.	4.23	0.601
	10. I often discuss various educational issues with colleagues and communicate with parents.	4.24	0.539
	11. I frequently communicate with parents to jointly solve various issues related to student development.	4.18	0.567
	12. I strive to garner more assistance and resources from parents and communities to achieve the school’s development goals.	4.11	0.677
	13. My words and actions can influence changes in parents.	4.02	0.693
Leadership Cognition	14. I have a very good understanding of teacher leadership.	3.84	0.762
	15. I possess teacher leadership.	3.82	0.727
	16. Teacher leadership behaviors are present in my school.	3.83	0.803

At the same time, we also noticed that in the dimension of “Leadership Cognition,” the scores for each item are not high, with average scores of only 3.84 points for “Understanding Teacher Leadership,” 3.82 points for “Having Leadership,” and 3.83 points for “Teacher Leadership Behavior Existence” items. Further analysis suggests that this might be due to the lack of a deep understanding of leadership concepts and practices among most teachers. Often, leadership is equated with managerial positions or administrative roles, without realizing that every teacher actually possesses potential leadership that can play a significant role in teaching, professional development, and school improvement.

In addition, influenced by traditional educational beliefs, some teachers may tend to position themselves as executors rather than decision-makers or leaders. They may be accustomed to receiving guidance from management rather than actively participating in school management and decision-making, thus limiting their own leadership development.

Regarding cross-cultural information technology teaching ability, the item “Valuing the cultivation of traditional Chinese culture and focusing on developing students’ multicultural abilities” scored 4.36, which not only demonstrates the firm belief and action of primary and secondary school teachers in inheriting and promoting traditional culture but also reflects their profound understanding that, in the context of global informationization, it is necessary not only to continue the inheritance of local culture but also to cultivate students’ global perspective and cross-cultural communication skills. Cultivating such abilities helps students better adapt to the challenges and opportunities brought about by globalization, and enhances international understanding and cooperation.

### 3.8. Deep learning

We conducted various data analyses, including reliability analysis and descriptive statistical analysis, on the collected data of leadership among primary and secondary school teachers in Xinjiang. These analyses provided insights into the current status and influencing factors of teacher leadership in the region from multiple perspectives. However, traditional data analysis methods only offer surface-level, linear relationship descriptions and cannot uncover deeper features and patterns behind leadership.

To delve into the characteristics of teacher leadership in Xinjiang, considering leadership as a complex, multi-level, and multi-dimensional concept, we decided to employ deep learning for further research.

Deep learning possesses powerful representation learning capabilities, automatically extracting useful features from raw data and revealing the complex structures behind the data through layered learning. Through the application of deep learning, we can explore and understand teacher leadership in primary and secondary schools in Xinjiang more thoroughly, providing more scientific and precise guidance for teacher leadership development. Deep learning can also assist in predicting teachers’ leadership levels. By training and optimizing deep learning models for teacher leadership, we may predict teachers’ leadership performance in different contexts, offering personalized training and development recommendations.

#### 3.8.1. Data source.

We processed and analyzed the questionnaire data using SPSS 26.0 software. To ensure the objectivity and accuracy of the data, we rigorously excluded questionnaires with continuous identical answers or consistently selecting the same response during the data cleaning process. This process aimed to eliminate potential risks and ensure the authenticity and reliability of the dataset, resulting in 371 valid questionnaires.

For an in-depth study of teacher leadership in primary and secondary schools, we collaborated with education experts with over 30 years of experience and holding senior professional titles. They participated in labeling and objectively analyzing the questionnaire data based on teachers’ performances in the five dimensions of professional guidance, home-school cooperation, leadership cognitive ability, social appeal, and cross-cultural informationized teaching ability. The leadership levels were categorized as “strong,” “average,” and “weak.”

After expert annotation, there were 82 teachers (22.1%) classified as “strong leadership,” 98 teachers (26.4%) as “average leadership,” and 192 teachers (51.7%) as “weak leadership”(The dataset is shown in S2 Data). The labeled data were then divided into training and testing sets in a 7:3 ratio, and all models used ten-fold cross-validation to measure the classification accuracy of different network models.

#### 3.8.2. Interpretable teacher leadership LSTM.

**Fig 1:**
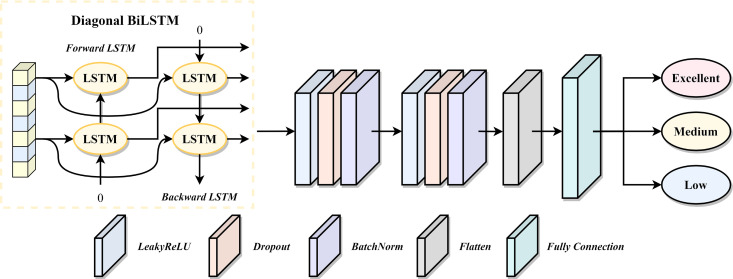
ITL-LSTM Structure.

In this context, we propose the ITL-LSTM (Interpretable Teacher Leadership LSTM) model, specifically designed for parsing and classifying the degree of teacher leadership. The model utilizes multiple Diagonal BiLSTM units, a structure capable of capturing long-term dependencies in sequential data, making it suitable for addressing continuous issues in questionnaires. With the deep bidirectional LSTM structure, the model can learn from both positive and negative directions, comprehensively understanding various dimensions of leadership. After dimensionality reduction using global average pooling layers, the model employs fully connected layers for classification output. The network structure is depicted in Fig 1.

Diagonal BiLSTM is a custom LSTM unit using a diagonal weight matrix structure, connecting each time step’s input and hidden state directly. In the leadership questionnaire data, specific questions have a significant impact on leadership levels, and the flexibility of the diagonal weight matrix better captures these features while reducing parameters. By stacking multiple Diagonal BiLSTM units, ITL-LSTM can learn more complex features and abstract representations, adapting to the multi-layer relationships and influencing factors that may exist in the data.

ITL-LSTM demonstrates excellent performance in handling leadership data. Its Diagonal BiLSTM structure is suitable for sequential data, effectively capturing the dynamic changes in leadership dimensions. The use of diagonal weight matrices reduces the number of parameters, enhancing generalization performance. The deep bidirectional LSTM structure accommodates the multi-level characteristics of the data. The application of global average pooling layers strengthens interpretability for leadership features. Overall, ITL-LSTM, with its robust temporal processing, interpretability, and efficiency, proves to have stronger classification capabilities for leadership level data.

#### 3.8.3. Model metrics.

In this study, the performance of the classification model is evaluated using metrics such as accuracy, sensitivity, specificity, and precision. The specific calculation formulas are as follows (1–4) and Table 10:

**Table 10:** Confusion Matrix.

	Positive	Negative
Positive	***TP***	***FP***
Negative	***FN***	***TN***


$$Accuracy=\frac{TP+TN}{TP+FP+TN+FN}$$ (1)

$$Sensitivity=\frac{TP}{TP+FN}$$ (2)

$$Specificity=\frac{TN}{TN+FP}$$ (3)

$$Precision=\frac{TP}{TP+FP}$$ (4)

#### 3.8.4. Deep learning result.

**Fig 2:**
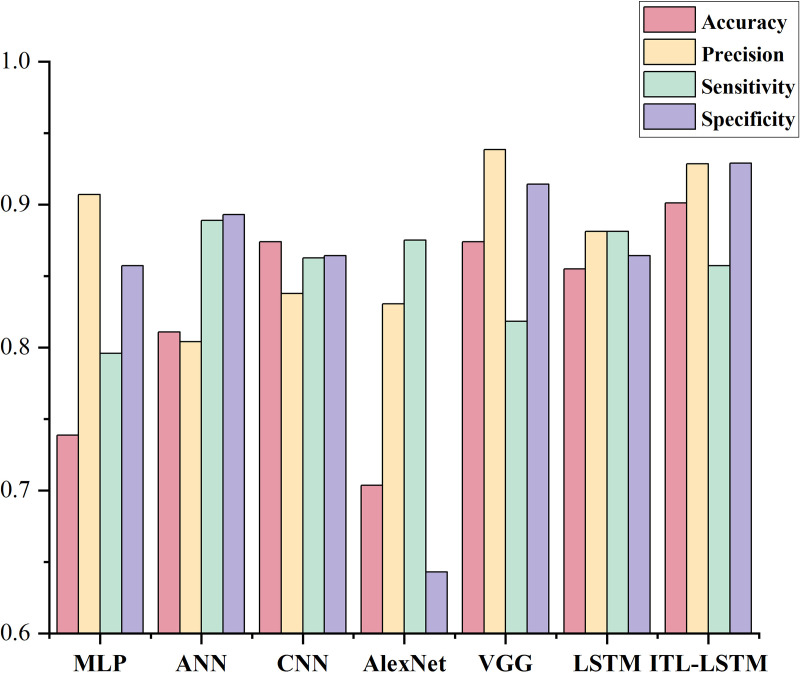
Experimental Results Comparison.

To enhance the robustness of the model and determine the optimal classifier, we adopted ten-fold cross-validation, using the mean evaluation of ITL-LSTM model performance as the final criterion. The results, as shown in Fig 2 and Table 11, reveal excellent performance by ITL-LSTM in the three-class task of leadership level, with an accuracy of 90.10%, precision of 92.85%, sensitivity of 88.11%, and specificity reaching 92.90%. Compared to traditional network models (MLP, ANN, CNN, AlexNet, VGG, LSTM), ITL-LSTM achieved higher accuracy by 16.23%, 9.01%, 2.71%, 19.74%, 2.71%, and 4.6%, respectively.

**Table 11:** ITL-LSTM Comparative Experimental Results.

Model	Accuracy	Precision	Sensitivity	Specificity
MLP	73.87%	90.70%	79.59%	85.71%
ANN	81.09%	80.4%	88.89%	89.29%
CNN	87.39%	83.78%	86.27%	86.43%
AlexNet	70.36%	83.05%	87.50%	64.29%
VGG	87.39%	93.83%	81.82%	91.43%
LSTM	85.50%	88.11%	88.11%	86.43%
**ITL-LSTM**	**90.10%**	**92.85%**	**85.71%**	**92.90%**

## 4. Discussion

This study focuses on the assessment and analysis of teacher leadership among primary and secondary school teachers in Xinjiang, aiming to address the gap in international research concerning leadership practices in multicultural and multi-ethnic educational contexts. While numerous studies have emphasized the significance of teacher leadership in advancing school development and improving student academic outcomes [25], most have been conducted in culturally homogeneous and resource-balanced settings, overlooking the unique dynamics of teacher leadership in pluralistic environments. This research attempts to bridge both theoretical and methodological gaps by integrating empirical investigation with a deep learning–based analytical model.

Regarding the influence of teacher leadership on student development, our findings align with those of Wang et al., affirming that strong teacher leadership contributes positively to students’ academic performance. In the context of Xinjiang, teachers exhibit notable strengths in guidance, organization, and cross-cultural communication, which not only enhance classroom efficiency but also expand students’ global perspectives and cultural tolerance through various intercultural activities. Compared with Chen’s (2022) findings from central China, our study reveals higher scores in the dimensions of “Cross-cultural Instructional Competence” and “Educational Collaboration,” suggesting greater adaptability among Xinjiang teachers to multilingual and multicultural environments.

From a methodological perspective, this study introduces the ITL-LSTM model, designed for dynamic modeling and prediction of teacher leadership. By incorporating a Diagonal BiLSTM structure, diagonal weight matrices, and global average pooling layers, the model effectively captures the temporal features and latent inter-dimensional relationships in leadership data. Compared with traditional static scale-based methods, the ITL-LSTM model not only achieves a classification accuracy of 90.10% but also offers superior interpretability and generalization, providing a more intelligent and precise tool for educational assessment.

A comparative review of existing teacher leadership assessment tools reveals three main limitations. First, current tools primarily rely on static measurement, lacking the capacity to capture behavioral dynamics—as seen in TLI scale [25] and Collective Leadership tool [26]. Second, most instruments are designed for specific regions or populations and thus lack adaptability to multi-ethnic and multilingual contexts [24,27], which are region-specific, or which targets only higher education [28], neglecting foundational education. Third, most tools are not integrated with advanced technologies such as artificial intelligence or deep learning, limiting their ability to predict and interpret leadership traits dynamically. The ITL-LSTM–based leadership assessment framework developed in this study systematically addresses these gaps by introducing a four-dimensional model encompassing Professional Guidance, Educational Collaboration, Cross-cultural Digital Instructional Competence, and Leadership Cognition. By combining this with a deep learning approach, the tool demonstrates strong scientific validity, contextual relevance, and predictive capacity.

To further enhance the contextual depth of our study, we supplemented our quantitative analysis with qualitative data from teacher interviews in Xinjiang. For instance, an English teacher from Changji noted: “Our students come from diverse backgrounds, and some struggle with Mandarin. I prepare bilingual vocabulary cards in advance and often ask bilingual classmates to help translate, so no one falls behind.” A rural teacher in Kashgar stated: “When resources are tight, whoever can lead will step up, even without an official title. We organize lessons, mentor newcomers—whatever is needed.” These frontline experiences illustrate how teachers interpret the dimensions of “Cross-cultural Instructional Competence” and “Educational Collaboration” in real practice, reinforcing the scale’s structural validity and the model’s predictive reliability. Additionally, a math department head from a key school in Ürümqi remarked: “Many young teachers are capable, but they don’t recognize themselves as leaders. I often remind them in meetings: if you’re guiding a lesson study group, you’re already leading.” This insight highlights the practical relevance of the “Leadership Cognition” dimension, reflecting how teachers gradually construct leadership awareness through practice.

## 5. Conclusions

This study developed and preliminarily validated a teacher leadership assessment tool tailored for multi-ethnic and multilingual educational environments. Based on survey data from 371 primary and secondary school teachers in Xinjiang, we proposed a four-dimensional framework encompassing Professional Guidance, Educational Collaboration, Cross-cultural Digital Instructional Competence, and Leadership Cognition. We further employed a Diagonal BiLSTM–based deep learning model—ITL-LSTM—to dynamically predict teacher leadership levels, achieving a classification accuracy of 90.10%.

The findings demonstrate that teacher leadership in multicultural educational contexts positively contributes to student motivation, cultural understanding, and classroom engagement. Teachers in Xinjiang, in particular, exhibited strong performance in cross-cultural pedagogy and collaborative practices, highlighting their adaptive capacity and leadership potential in complex educational environments.

From a practical perspective, this study provides education administrators and policymakers with an operational tool for identifying leadership development pathways and optimizing professional growth strategies. At the international level, the proposed assessment framework and modeling approach offer transferable value for teacher leadership research in other multicultural and resource-diverse settings.

Nevertheless, several limitations should be acknowledged. Although Xinjiang serves as a highly representative case, the generalizability of the findings to the national or global level requires further validation. While the study emphasizes the practical dimensions of teacher leadership, data collection relied primarily on quantitative measures, and qualitative insights remain relatively limited. Additionally, the model development focused on tool construction, without fully exploring the dynamic interplay between leadership growth trajectories and institutional contexts. Future research may extend the sample scope to include broader geographic and cultural backgrounds, refine model parameters, and explore its integration into dynamic feedback and intervention systems—thereby supporting equitable education and sustained professional development in a broader context.

## Supporting information

S1 Data Orginal data. (XLSX)

S2 Data All_data. (XLSX)
